# Curvature‐Controlled Field Effect Enables Thermal Localization for Low‐Temperature C─F Bond Activation

**DOI:** 10.1002/advs.76372

**Published:** 2026-06-28

**Authors:** Hang Zhang, Jialin Zheng, Xiaojian Wang, Hao Yu, Diya Xie, Wenjie Luo, Minghui Yang, Kang Liu, Yuxia Duan, Zhang Lin, Liyuan Chai, Emiliano Cortés, Min Liu

**Affiliations:** ^1^ Hunan Joint International Research Center For Carbon Dioxide Resource Utilization School of Physics Central South University Changsha Hunan China; ^2^ Nanoinstitute Munich Faculty of Physics Ludwig‐Maximilians‐Universität München München Germany; ^3^ School of Metallurgy and Environment Central South University Changsha Hunan China

**Keywords:** carbon tetrafluoride (CF_4_), C─F bond activation, curvature‐controlled field effect, localized thermal fields

## Abstract

Geometric singularities are known to concentrate electric and optical fields, but whether curvature alone can localize thermal energy and thereby influence chemical kinetics remains unresolved. Here, we show experimentally and computationally that nanoscale curvature generates localized thermal fields that directly lower reaction barriers in heterogeneous catalysis. Using γ‐Al_2_O_3_ architectures with systematically varied curvature, nanoneedles, micro‐needles, and spheres, while maintaining comparable composition, phase, acidity, and defect states, we observe curvature‐dependent temperature localization of up to ∼30 °C at nanoneedle tips under identical external heating. In situ infrared thermography confirms hotspot formation, and operando vibrational spectroscopy reveals enhanced water dissociation and increased *CF_3_ intermediate populations during CF_4_ decomposition. These effects reduce the apparent activation energy and enable complete CF_4_ decomposition at 580 °C, substantially below that required for lower‐curvature structures. The results establish curvature‐induced thermal localization as a general physical mechanism linking geometry to interfacial energy density and reaction kinetics, providing a universal design principle for activating strongly bound molecules.

## Graphical abstract

High‐curvature γ‐Al_2_O_3_ nanoneedles concentrate heat into nanoscale hotspots under identical external heating, amplifying local temperature and accelerating CF_4_ decomposition. By linking curvature‐induced thermal localization to lower apparent activation barriers and enhanced intermediate formation, this work reveals geometry as a powerful design parameter for activating chemically resilient molecules.

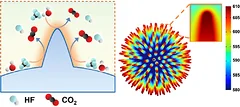

## 1 Introduction

Control of local energy density at interfaces is fundamental to surface reaction kinetics [1, 2, 3]. Conventional strategies tune electronic structure [4, 5], defects [6, 7], or chemical composition [8, 9] to reduce activation barriers, implicitly assuming that temperature remains spatially uniform at the catalyst surface [10]. However, geometry itself can concentrate physical fields. Sharp features are well known to amplify electric, optical, and electrochemical responses through field localization [11, 12, 13]. Whether an analogous curvature‐driven localization of thermal energy can arise under uniform external heating, and whether such localization can directly regulate chemical kinetics, remains largely unexplored [14, 15, 16].

This question becomes particularly important for reactions involving exceptionally strong chemical bonds. Carbon tetrafluoride (CF_4_) is among the most stable fluorocarbons [17, 18, 19], possessing one of the highest C─F bond dissociation energies in small molecules and requiring high temperatures for decomposition [20, 21, 22, 23, 24]. Existing catalytic strategies mainly rely on modifying alumina surface chemistry, yet even optimized systems typically demand temperatures of 650°C or higher for full conversion [25, 26, 27, 28]. Demonstrating that geometry alone can lower this threshold would reveal a fundamentally different route for controlling reaction kinetics based on physical energy localization rather than chemical modification.

Here, we isolate curvature as the governing parameter by synthesizing γ‐Al_2_O_3_ catalysts with matched phase, electronic structure, and surface chemistry but systematically different radii of curvature: spherical particles (low curvature), micro‐needles (intermediate curvature), and nanoneedles (high curvature). Combining heat‐transfer simulations, direct infrared thermography, operando spectroscopy, and kinetic measurements, we show that increasing curvature produces localized thermal hotspots that enhance molecular activation and reduce reaction barriers. These results provide direct experimental evidence that curvature‐induced thermal field localization can control catalytic performance and establish geometry as an intrinsic parameter governing interfacial kinetics.

## 2 Results and Discussion

### 2.1 Curvature‐Dependent Thermal Localization

Finite‐element simulations (COMSOL Multiphysics) were used to evaluate steady‐state temperature distributions for alumina structures subjected to identical external heating (Figure 1a and Figure S1) [29, 30, 31]. Spherical particles exhibited nearly uniform surface temperatures with fluctuations below ∼5 °C (Figure 1b). Micro‐needle geometries produced moderate localization (∼15 °C enhancement) (Figure 1c), whereas nanoneedle tips generated pronounced hotspots approaching ∼30 °C above surrounding regions (Figure 1d). The simulations indicate that increasing curvature alters heat‐flux convergence and dissipation, leading to spatially confined temperature amplification.

**FIGURE 1:**
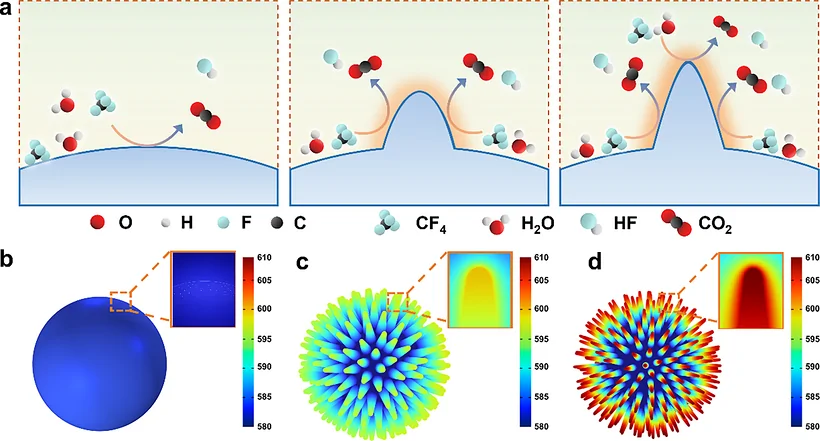
(a) Schematic illustration of thermal field distributions during CF_4_ catalytic hydrolysis over catalysts with varying curvature radii. The local surface temperature distribution simulated by COMSOL for (b) low‐curvature Al_2_O_3_ spherical, (c) medium‐curvature Al_2_O_3_ micro‐needle, and (d) high‐curvature Al_2_O_3_ nanoneedle catalysts during the catalytic hydrolysis of CF_4_.

### 2.2 Isolation of Geometry as the Dominant Variable

Guided by the simulations, we synthesized three γ‐Al_2_O_3_ catalysts with well‐controlled morphologies: nanoneedle, micro‐needle, and spherical (Figure 2a). X‐ray diffraction confirmed the γ‐Al_2_O_3_ phase for all samples (Figure S2). SEM and TEM imaging revealed urchin‐like microspheres composed of nanoneedles or micro‐needles (∼2 µm diameter), while smooth spherical particles were obtained via a sol–gel route (Figure 2b–d and Figures S3–S5).

**FIGURE 2:**
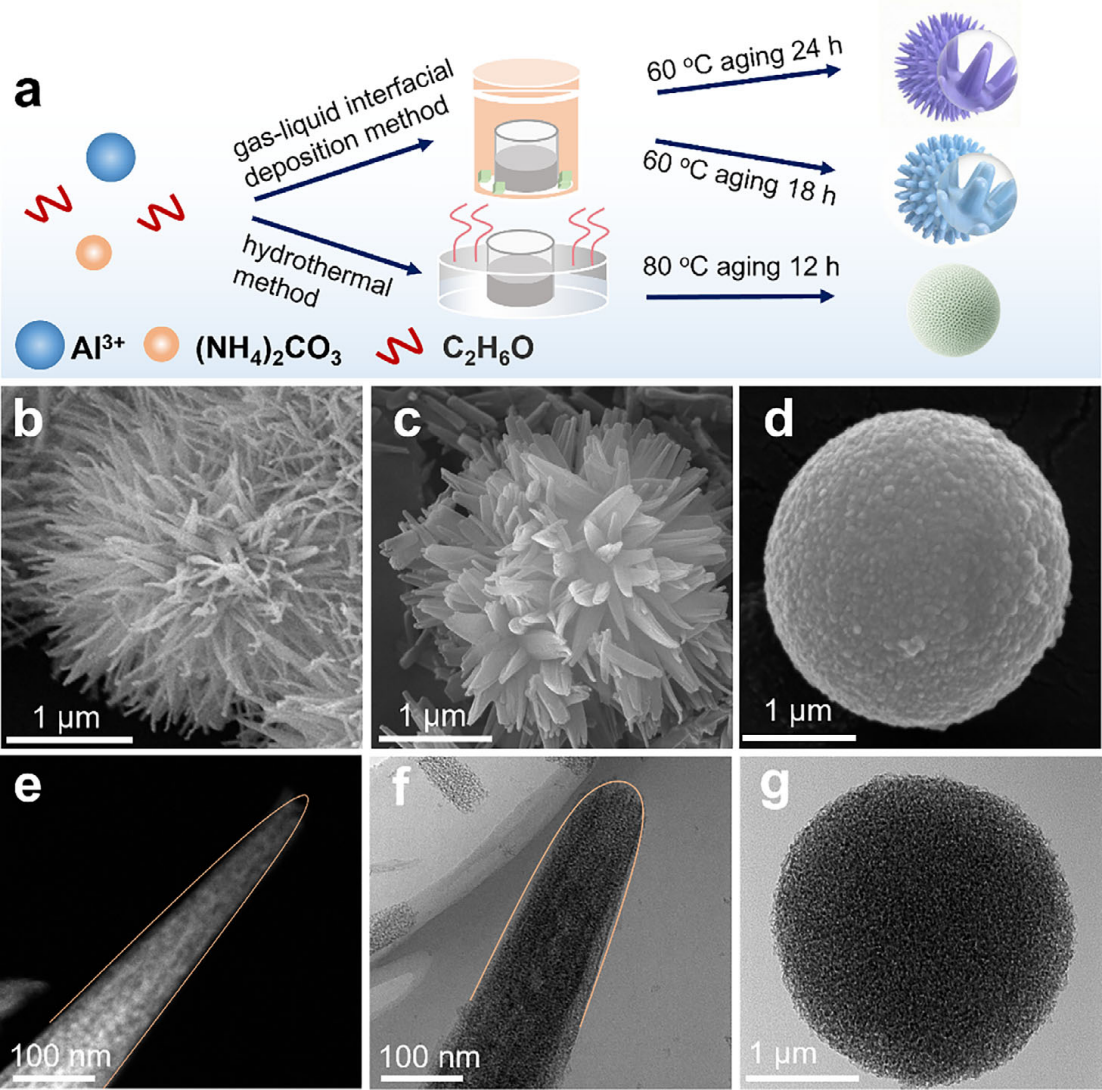
(a) Schematic illustration of catalyst preparation. SEM images of (b) Al_2_O_3_ nanoneedle, (c) Al_2_O_3_ micro‐needle, and (d) Al_2_O_3_ spherical. TEM images of (e) Al_2_O_3_ nanoneedle, (f) Al_2_O_3_ micro‐needle, and (g) Al_2_O_3_ spherical catalysts.

HRTEM showed nearly identical lattice spacings (∼0.137–0.138 nm), corresponding to the γ‐Al_2_O_3_ (110) plane (Figure S6). Elemental mapping confirmed uniform Al and O distribution (Figure S7). BET measurements indicated comparable surface areas (259.7, 248.2, and 268.6 m^2^ g^−1^ for nanoneedle, micro‐needle, and spherical catalysts) and similar mesoporous structures (Figures S8 and S9, and Table S1). NH_3_‐TPD and pyridine‐IR showed that acidity does not correlate with activity, and XPS analysis confirmed similar electronic structures and defect states (Figures S10–S13). These results indicate that the observed performance differences originate primarily from curvature‐controlled physical effects rather than compositional or chemical changes. CF_4_‐TPD measurements further revealed stronger adsorption on the nanoneedle catalyst, with ∼1.5‐fold higher uptake than the spherical sample (Figure S14), consistent with enhanced local activation environments [32].

### 2.3 In Situ Infrared Thermography

To experimentally validate curvature‐dependent thermal localization, in situ infrared thermography was performed in a fixed‐bed reactor [33, 34, 35, 36]. All catalysts showed identical baseline temperatures prior to heating (Figure 3a,b). Upon heating, their surface temperatures diverged systematically.

**FIGURE 3:**
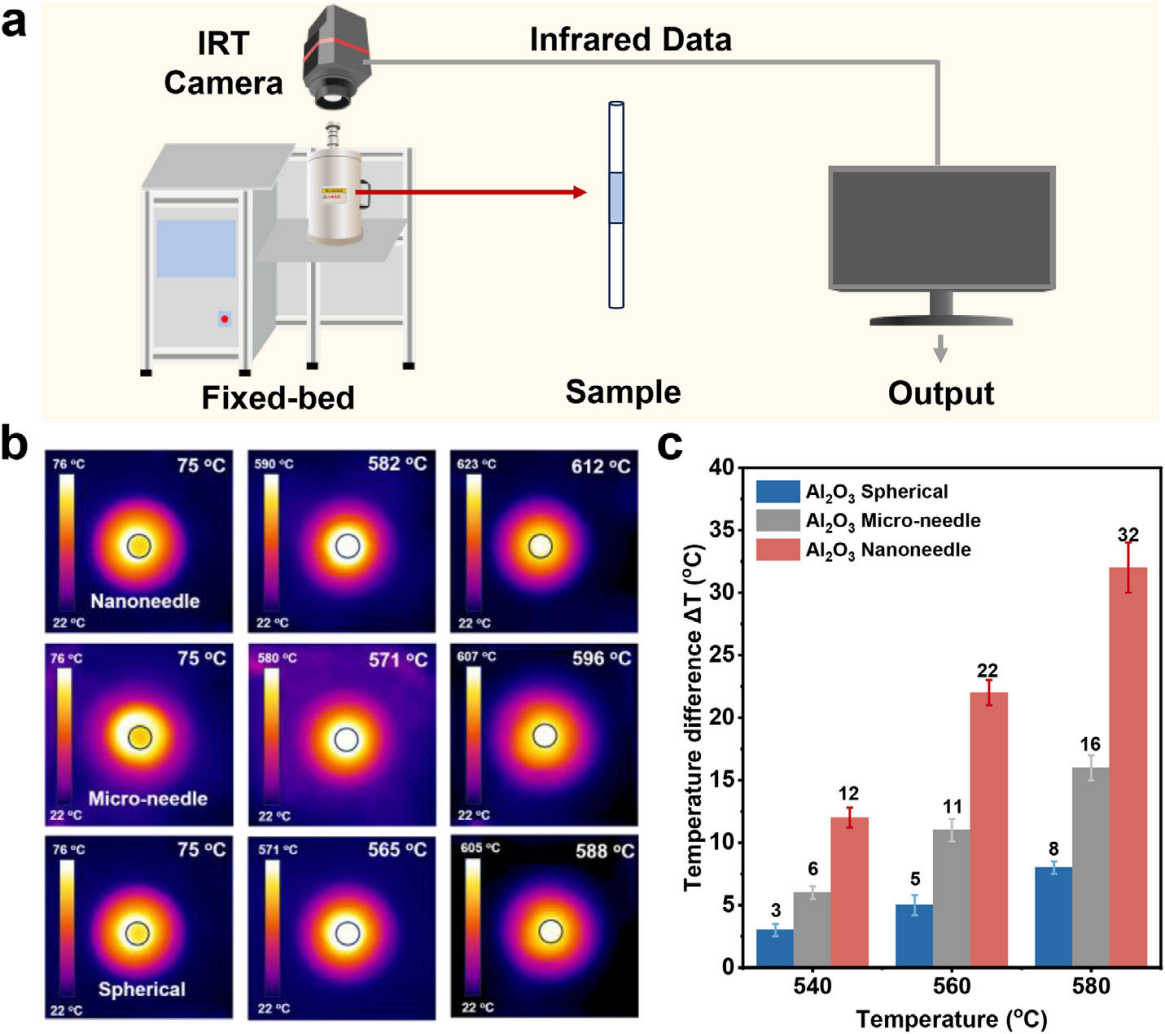
(a) Schematic diagram of infrared thermal imaging test. (b) Thermal infrared thermography of Al_2_O_3_ nanoneedle, Al_2_O_3_ micro‐needle, and Al_2_O_3_ spherical catalysts. (c) Temperature‐difference graphs of the experimental heating results of the Al_2_O_3_ nanoneedle, Al_2_O_3_ micro‐needle, and Al_2_O_3_ spherical catalysts.

At nominal reactor temperatures of 540 °C, 560 °C, and 580 °C, the nanoneedle catalyst reached 552 °C, 582 °C, and 612 °C, respectively. Under identical conditions, the micro‐needle catalyst reached 546 °C, 571 °C, and 596 °C, while the spherical catalyst reached 543 °C, 565 °C, and 588 °C. At 580 °C, the temperature enhancements were approximately 32 °C, 16 °C, and 8 °C, respectively (Figure 3c and Figure S15). Although infrared thermography provides relative values, the consistent curvature‐dependent divergence confirms localized thermal enrichment, in agreement with simulations. The threshold for this thermal localization effect was further examined by infrared thermal imaging from 75 °C to 580 °C (Figure S16). The localized thermal field became observable at approximately 250 °C and intensified with increasing external heating temperature. Since this threshold is much lower than the temperature required for CF_4_ catalytic hydrolysis, the tip‐induced thermal effect is already effective under the reaction conditions, supporting its role in facilitating molecular activation.

To quantitatively define the curvature of the three catalysts, the radius of curvature at the representative terminal region was measured from TEM images, and the curvature was calculated according to κ = 1/r, where κ is the curvature and r is the radius of curvature. The calculated curvatures of the Al_2_O_3_ spherical, micro‐needle, and nanoneedle catalysts are approximately 0.001, 0.02, and 0.05 nm^−1^, respectively. By correlating these curvature values with the thermal‐field enhancement obtained from infrared thermal imaging, a clear positive relationship was observed at nominal fixed‐bed temperatures of 540 °C, 560 °C, and 580 °C (Figure S18). These results quantitatively confirm that the localized thermal field is strongly dependent on the curvature magnitude of the catalyst.

### 2.4 Operando Evidence of Enhanced Molecular Activation

In situ DRIFTS measurements were performed from 100 °C to 580 °C (Figure 4a). All catalysts exhibited depletion of surface Al–OH groups (negative band at 3760 cm^−1^), indicating water participation in hydrolysis [37, 38]. This depletion was strongest for the nanoneedle catalyst, suggesting enhanced surface reactivity (Figure S19).

**FIGURE 4:**
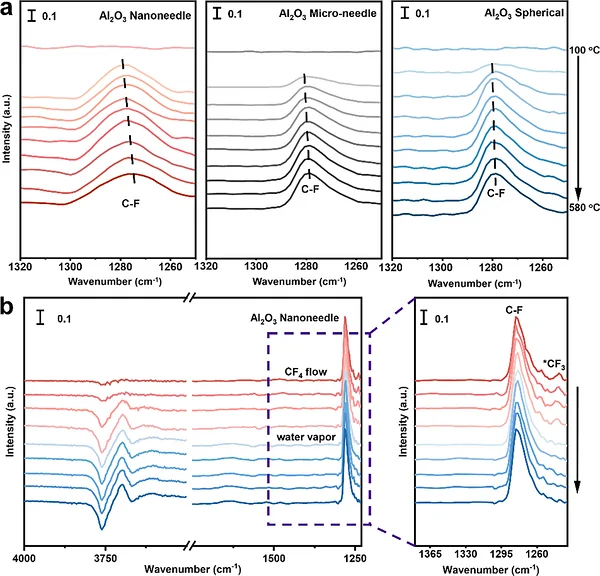
In situ DRIFTS of the CF_4_ catalytic hydrolysis. (a) In situ DRIFTS over Al_2_O_3_ nanoneedle catalyst, Al_2_O_3_ micro‐needle catalyst, and Al_2_O_3_ spherical catalysts within the temperature range of 100 °C–580 °C. (b) Al_2_O_3_ nanoneedle catalyst was first exposed to CF_4_ alone, and then H_2_O was introduced at 580 °C.

The C─F stretching band (∼1280 cm^−1^) shifted progressively to lower frequency with increasing curvature, reaching 1275 cm^−1^ for the nanoneedle catalyst, indicating stronger C─F bond activation [39]. Additional bands at 2320 cm^−1^ (CO_2_), 1640 cm^−1^ (water dissociation), and 1250 cm^−1^ (*CF_3_ intermediate) were significantly stronger for the nanoneedle catalyst (Figures S20 and S21) [40]. Control experiments under anhydrous conditions confirmed *CF_3_ formation (Figure 4b), while introducing water rapidly consumed this species, consistent with hydroxyl‐mediated hydrolysis pathways.

### 2.5 Kinetic Consequences of Curvature‐Induced Hotspot

Catalytic testing showed that the nanoneedle catalyst consistently outperformed lower‐curvature structures (Figure 5a). At a nominal fixed bed temperature of 580 °C, the nanoneedle catalyst achieved complete CF_4_ conversion, whereas the micro‐needle and spherical catalysts reached 80% and 63%, respectively. The complete‐conversion temperatures of the micro‐needle and spherical catalysts were further determined to be 600 °C and 610 °C, respectively. These results confirm that the high‐curvature nanoneedle catalyst enables complete CF_4_ conversion at a substantially reduced temperature.

**FIGURE 5:**
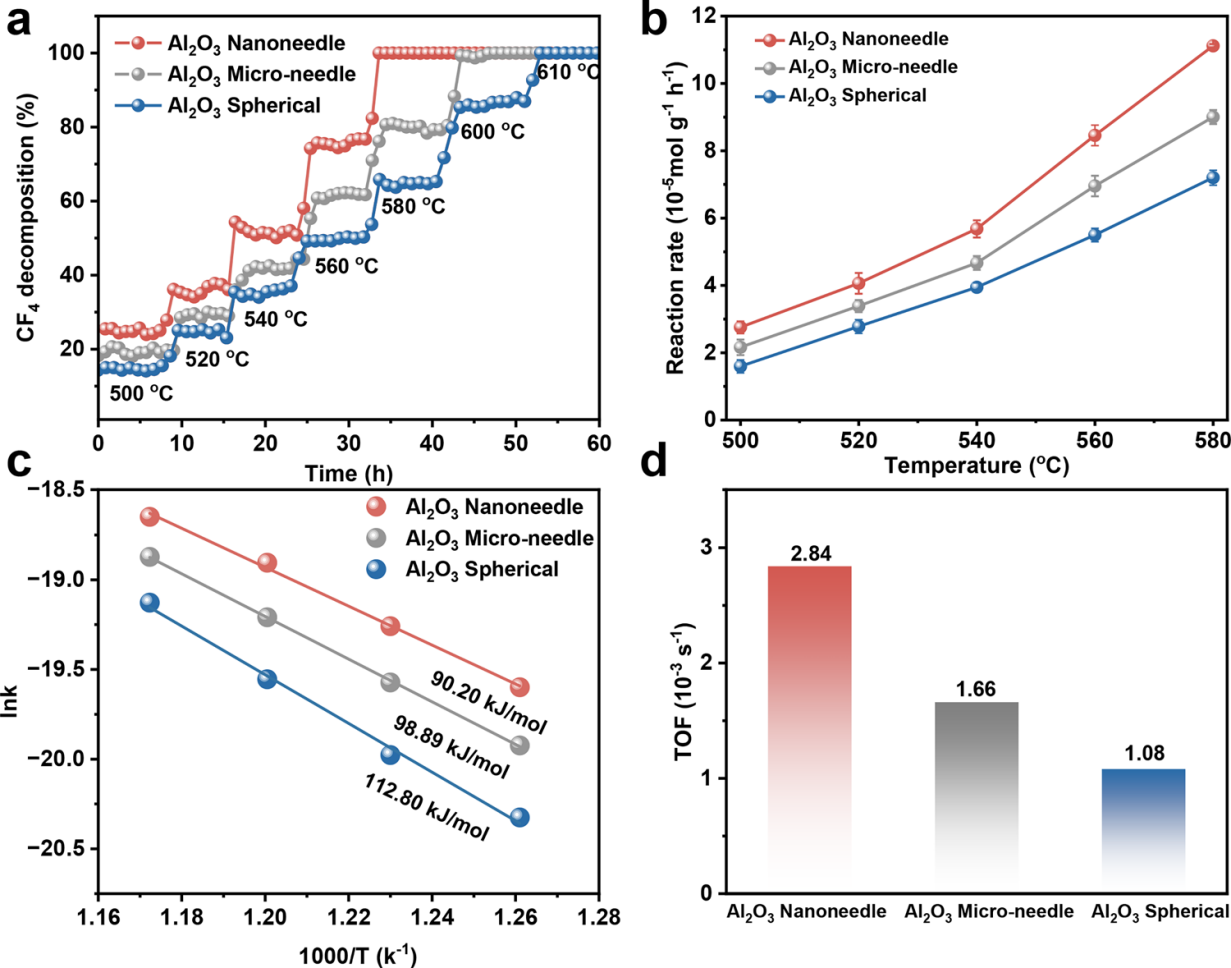
(a) CF_4_ decomposition rate (%) of catalytic hydrolysis of CF_4_ by Al_2_O_3_ nanoneedle, Al_2_O_3_ micro‐needle, and Al_2_O_3_ spherical catalysts at different nominal fixed bed temperatures. (b) Reaction rate of Al_2_O_3_ nanoneedle, Al_2_O_3_ micro‐needle, and Al_2_O_3_ spherical catalysts at different reaction temperatures. (c) Arrhenius plots of CF_4_ decomposition rates for the Al_2_O_3_ nanoneedle, Al_2_O_3_ micro‐needle, and Al_2_O_3_ spherical catalysts (500 °C–580 °C). (d) TOF calculation for CF_4_ catalytic decomposition over Al_2_O_3_ nanoneedle, Al_2_O_3_ micro‐needle, and Al_2_O_3_ spherical catalysts.

Reaction rates at 580 °C were 11.2 × 10^−5^, 8.5 × 10^−5^, and 7.2 × 10^−5^ mol g^−1^ h^−1^ for nanoneedle, micro‐needle, and spherical catalysts (Figure 5b). Arrhenius analysis yielded apparent activation energies of 90.2, 98.9, and 112.8 kJ mol^−1^, respectively (Figure 5c). TOF values normalized to acidity followed the same trend (Figure 5d). Notably, although the nanoneedle catalyst shows only a 1.5‐fold increase in CF_4_ adsorption compared with the spherical catalyst, its TOF is enhanced by about 2.6 times. This indicates that the dominant role of curvature‐induced localized thermal‐field‐assisted C─F bond activation. Long‐term testing demonstrated stable nanoneedle performance for over 120 h at 560 °C (Figure S22).

## 3 Conclusion

In this study, we demonstrate that nanoscale curvature intrinsically localizes thermal energy and reduces reaction barriers in heterogeneous catalysis. γ‐Al_2_O_3_ nanoneedles generate localized hotspots of ∼30 °C, confirmed by in situ thermography and simulations. Operando spectroscopy shows that these hotspots enhance water dissociation and CF_3_ intermediate formation during CF_4_ hydrolysis, lowering activation energy and enabling complete conversion at substantially reduced temperature. These findings establish curvature‐induced thermal localization as a general physical mechanism connecting geometry to interfacial kinetics and provide a broadly applicable strategy for activating strongly bound molecules through structural design alone.

## 4 Experimental Section

### 4.1 Chemicals

All chemicals were obtained commercially and used as received. Ammonium carbonate ((NH_4_)_2_CO_3_, AR), aluminum chloride hexahydrate (AlCl_3_·6H_2_O, 97%), propylene oxide (PO, 99%), anhydrous ethanol (CH_3_CH_2_OH, ≤0.3%), and aluminum nitrate nonahydrate (Al(NO_3_)_3_∙9H_2_O, 99%) were purchased from Aladdin.

### 4.2 Materials Preparation

#### 4.2.1 Synthesis of Al_2_O_3_ Nanoneedle

Preparation of catalyst precursors by gas‐liquid interfacial deposition method [41]. First, 3.75 g of Al(NO_3_)_3_·9H_2_O was dissolved in 100 mL of deionized water and stirred until completely dissolved. Then, the beaker containing the solution was transferred to a sealed container. Next, 3.84 g of (NH_4_)_2_CO_3_ powder was evenly placed around the beaker (molar ratio of Al^3+^: NH_4_^+^ was 1:8). The sealed container was placed in a 60 °C oven for a reaction period of 24 h. After the reaction, the precipitate was collected and washed with deionized water (3 times) and with anhydrous ethanol (2 times) to remove impurities. The obtained product was dried in a 60 °C oven. Finally, the dried precursor was calcined in air at 600 °C for 5 h to obtain the target catalyst.

#### 4.2.2 Synthesis of Al_2_O_3_ Micro‐Needle

The Al_2_O_3_ micro‐needle catalyst was prepared using the same gas‐liquid interfacial deposition method as that employed for the nanoneedle catalyst. The only difference is that the aging time for the micro‐needle structure was adjusted to 18 h.

#### 4.2.3 Synthesis of Al_2_O_3_ Spherical

Catalyst precursor synthesis via sol‐gel method [29]: 7.24 g of AlCl_3_·6H_2_O was dissolved in a mixed solvent comprising 6 mL deionized water and 9 mL anhydrous ethanol under vigorous magnetic stirring at 80 °C until complete dissolution. Subsequently, 10.5 mL of PO was added dropwise to this solution as a gelation agent. After complete addition, the obtained solution was sealed and kept in an oven at 80 °C for 12 h for gelation. Finally, the wet gel was placed in a muffle furnace and calcined at 600 °C for 5 h at a heating rate of 3 °C/min in an air atmosphere to obtain the target catalyst.

### 4.3 Catalytic Activity Evaluation

Under normal pressure conditions, the catalytic hydrolysis reaction of CF_4_ was evaluated in a continuous‐flow fixed‐bed quartz reactor system. A 2.0 g catalyst bed was packed in the reactor. Reaction temperatures were varied from 500 °C to 600 °C. The gaseous feed (33.3 mL min^−1^ total flow), controlled by mass flow controllers, contained 0.25 vol% CF_4_ balanced in argon. Deionized water (0.8 mL h^−1^) was introduced via syringe pump, vaporized in a pre‐heated chamber (200 °C), and mixed with the main gas stream upstream of the reactor inlet.

$$CF_{4}\;\mathrm{decomposition}\;\left(\%\right)=\frac{{\left[CF_{4}\right]}_{\mathrm{in}}-{\left[CF_{4}\right]}_{\mathrm{out}}}{{\left[CF_{4}\right]}_{\mathrm{in}}}\;\times 100\%$$

where [CF_4_]_in_ and [CF_4_]_out_ indicate the inlet and outlet relative concentrations, respectively.

### 4.4 Catalyst Characterization

X‐ray powder diffraction (XRD) was measured using a Bruker D8 Focus diffractometer and scanning in the 2θ range of 10 to 90° with Cu Kα (40 kV, 40 mA) radiation as the X‐ray source.

Transmission electron microscopy (TEM, JEOL 3010, 200 kV) and scanning electron microscopy (SEM, MIRA3 LMH, 20.0 kV) were employed to characterize the sample morphology. For TEM observation, the sample was dispersed in ethanol and dropped onto a copper grid. Prior to SEM imaging, the sample was coated with a gold layer.

X‐ray photoelectron spectroscopy (XPS) analysis was conducted on a Kratos Axis Ultra DLD spectrometer equipped with a monochromatic Al Kα source (1486.6 eV), using a pass energy of 40 eV. All binding energies were calibrated against the adventitious carbon (C *1s*) peak at 284.8 eV.

N_2_ adsorption‐desorption isotherms were acquired on a Micromeritics ASAP 2460 system to quantify the specific surface area and pore size distribution of the catalysts. Samples were degassed at 300°C for 6 h prior to analysis.

NH_3_ Temperature programmed desorption (NH_3_‐TPD) was measured with a PCA‐1200 chemical absorption analyzer equipped with a TCD detector. The catalyst, in the weight of 0.1 g, underwent pretreatment first at 600 °C for 2 h in Ar flow, to remove surface impurities and moisture from the sample. Then, switch the gas to NH_3_ for 1 h, followed by purging with Ar gas for 1 h at room temperature. Upon removing the physically adsorbed NH_3_, begin heating at a rate of 10 °C min^−1^ up to 600 °C.

CF_4_ temperature‐programmed desorption (CF_4_‐TPD) was conducted on a TP5080 automatic multipurpose adsorption apparatus equipped with a thermal conductivity detector (TCD). 0.1 g of catalyst was pretreated under Ar at 600 °C for 2 h. The sample was then saturated with CF_4_ at ambient temperature for 1 h, followed by Ar purging for 1 h to eliminate physically adsorbed species. Finally, temperature‐programmed desorption was performed from 25 °C to 600 °C at a heating rate of 10 °C·min^−1^ under Ar flow (30 mL·min^−1^).

To ensure scientific rigor and interpretive reliability, we conducted in situ temperature monitoring under true fixed‐bed reaction conditions (with 2.0 g catalyst loading). Prior to the formal measurements, we calibrated the emissivity of catalysts. During the fixed‐bed reaction tests, the IR camera was positioned vertically above the catalyst bed to enable real‐time monitoring, and a thermocouple was placed at the same location to provide simultaneous temperature readings. Specifically, the emissivity of all three Al_2_O_3_ samples was set to 0.68 during infrared thermal imaging. The measurement distance between the infrared camera and the catalyst bed was 20 cm. The thermocouple was positioned in the middle of the catalyst bed to monitor the nominal fixed‐bed temperature (Figure S15). Each measurement was repeated three times. By comparing the thermocouple and IR measurements, we adjusted the emissivity parameters for each catalyst individually, thereby minimizing potential temperature deviations caused by emissivity differences.

In situ diffuse reflectance infrared Fourier transform spectroscopy (DRIFTS) was performed using a Thermo Fisher iS50 spectrometer, acquiring spectra from 800 to 4000 cm^−1^. The catalyst sample was first loaded into an in situ reaction cell and pretreated under 30 mL·min^−1^ Ar flow at 300 °C for 1 h. Following pretreatment, the reaction was initiated by introducing CF_4_ and H_2_O vapor, with signal evolution monitored in real‐time. Water vapor is introduced through a deionized water bottle.

### 4.5 COMSOL Simulations

The finite element method (FEM) simulations were conducted through COMSOL Multiphysics v 6.2. To explore the performance changes caused by the effects of catalysts with different appearances on thermal fields, the Heat Transfer (HT) physics modules in COMSOL Multiphysics were used to simulate and calculate the performance of the models. The experimental images were used to model the catalyst particles, and two shapes were established: spherical and sea urchin. All three catalyst models are ∼2 µm in diameter. The needle‐shaped model has a 500 nm tip with a curvature of 0.05 nm^−1^, whereas the micro‐needle model has a shorter 400 nm tip and a lower curvature of 0.01 nm^−1^. All the meshes in the model were set to free tetrahedral meshing. The relative tolerance in the steady‐state solver was set to 0.0001.

Considering the reaction carried out by the system as an exothermic reaction:

$$CF_{4}+2H_{2}O\to CO_{2}+4HF\;\left(\Delta H=-168.1\;kJ\;mol^{-1}\right)$$

where Δ*H* is calculated based on bond energy and is temperature‐corrected using the heat capacity at constant pressure

$$\Delta H=\int_{T_{ustr}}^{T}C_{p}dT$$

The reactants in the reaction system are CF_4_ and H_2_O, the products are CO_2_ and HF, and the carrier gas is Ar.

The heat generated by the catalyst particles is estimated using the following formula:

$$Q_{total}=flow\;rate_{air+CF_{4}}\;\times 0.25\%\times \frac{RT}{P}\times \Delta H$$

and $Q_{i}=\frac{Q_{total}}{n_{i}}$where $flow\;rate_{air+CF_{4}}\times 0.25\%$ represents the gas flow rate entering the reactor under operating conditions and the proportion of CF_4_, where *n_i_* denotes the number of catalyst particles estimated.

Considering both heat conduction and convection, the specific formula is:

$$\rho C_{p}u\cdot \nabla T+\nabla \cdot q\;=Q+Q_{p}+Q_{vd}$$

where *C_p_* is the heat capacity at constant pressure, *T* is the thermodynamic temperature, ρ is the material density, *u* is the velocity vector, Q, *Q_p_* and *Q_vd_* are the heat source terms, ∇ · q is the divergence of the temperature gradient. It can be obtained from the following formula:

$$q\;=-k_{iso}\nabla T$$

where *k_iso_* is the thermal conductivity of the material.

The gas thermal conductivity is corrected using the following formula:

$$\begin{array}{ccc} k_{iso,\gamma -Al_{2}O_{3}} & = & -0.00227583562+1.15480022\times 10^{-4}\\ & & \times \;T-7.90252856\times 10^{-8}\times T^{2}+4.11702505\\ & & \times \;10^{-11}\times T^{3}-7.43864331\times 10^{-15}*T^{4} \end{array}$$

The density of the catalyst particles is corrected using the following formula:

$$\rho_{\gamma -Al_{2}O_{3}}=\left(3726.112-0.0896537*T\right)$$

The boundary conditions for the inflow and outflow of the gas flow field are described by the following equations:

−n·q=ρΔHu·n

where *n* is the direction vector. The boundary temperature is set to the reaction temperature *T*_0_.

The gas flow rate through the catalyst particles is corrected by the porosity ε_*i*_ and is calculated in conjunction with the reactor radius:

$$\begin{array}{ccc} flow\;rate_{total} & = & flow\;rate_{air+CF_{4}}+flow\;rate_{water}\\ & = & \frac{TP_{0}*33.3\;sccm}{T_{0}P}\\ & & +\left(\frac{0.06\;g}{M_{H_{2}O}}*\frac{RT}{P}*10^{6}\;\frac{cm^{3}}{m^{3}}\right)\;s^{-1} \end{array}$$

and $v_{total}=\frac{flow\;rate_{total}}{\pi {r_{reactor}}^{2}\left(\varepsilon_{i}\right)}$where *r_reactor_* is the reactor radius. ε_*i*_ is estimated based on the experimental bulk density of the catalyst:

$$\varepsilon_{i}=1-\frac{\rho_{bulk\;i}}{\rho_{\gamma -Al_{2}O_{3}}}$$

## Author Contributions

**Hang Zhang**: conceptualization, investigation, writing – original draft, visualization, formal analysis. **Hao Yu**: investigation, methodology, writing – review and editing, formal analysis. **Jialin Zheng**: investigation, writing – original draft, methodology, validation, visualization, formal analysis. **Wenjie Luo**: investigation, methodology, writing – review and editing, formal analysis. **Yuxia Duan**: investigation, writing – review and editing, formal analysis. **Min Liu**: supervision, resources, project administration, writing – original draft, conceptualization, funding acquisition. **Minghui Yang**: investigation, writing – review and editing, visualization, formal analysis. **Kang Liu**: methodology, writing – review and editing, formal analysis. **Liyuan Chai**: writing – review and editing, data curation, formal analysis. **Diya Xie**: investigation, writing – review and editing, visualization, formal analysis. **Emiliano Cortés**: supervision, resources, writing – original draft, conceptualization, funding acquisition. **Xiaojian Wang**: investigation, writing – review and editing, methodology, formal analysis. **Zhang Lin**: data curation, writing – review and editing, formal analysis.

## Conflicts of Interest

The authors declare no conflicts of interest.

## Supporting information




**Supporting File**: advs76372‐sup‐0001‐SuppMat.docx.

## Data Availability

The data that support the findings of this study are available from the corresponding author upon reasonable request.
