# Supplementary material for: Curvature‐Controlled Field Effect Enables Thermal Localization for Low‐Temperature C─F Bond Activation
Source: Adv Sci (Weinh). 2026 Jun 28;13(53):e76372. doi: 10.1002/advs.76372 (PMC13336511; doi:10.1002/advs.76372)
Supplement: Supplementary file 1 [file ADVS-13-e76372-s001.docx]

**Curvature-Controlled Field Effect Enables Thermal Localization for Low-Temperature C–F Bond Activation**

Hang Zhang,^†[a,c]^ Jialin Zheng,^†[a,b]^ Xiaojian Wang,^†[a,b]^ Hao Yu,^[a]^ Diya Xie,^[c]^ Wenjie Luo,^[a]^ Minghui Yang,^[a]^ Kang Liu,^[a]^ Yuxia Duan,^[a]^ Zhang Lin^[b]^, Liyuan Chai^[b]^, Emiliano Cortés,^*[c]^ and Min Liu^*[a,b]^

[a] Hunan Joint International Research Center for Carbon Dioxide Resource Utilization, School of Physics, Central South University, Changsha 410083, Hunan, China.

[b] School of Metallurgy and Environment, Central South University, Changsha 410083, Hunan, China.

[c] Nanoinstitute Munich, Faculty of Physics, Ludwig-Maximilians-Universität München, 80539, München, Germany.

[†] H.Z., J.Z., and X.W. contributed equally

*Min Liu and Emiliano Cortés.

Email: minliu@csu.edu.cn; Emiliano.Cortes@lmu.de

**Supplementary Figure 1 |** Different temperature distribution models of nanoneedle catalysts with high curvature. The reaction environment is set at 580 °C.

**Supplementary Figure 2 |** XRD patterns of the Al_2_O_3_ nanoneedle, micro-needle, spherical catalysts.

**Supplementary Figure 3 |** SEM image of Al_2_O_3_ nanoneedle catalyst.

**Supplementary Figure 4 |** TEM image of Al_2_O_3_ micro-needle catalyst.

**Supplementary Figure 5 |** TEM images of Al_2_O_3_ spherical catalyst.

**Supplementary Figure 6 |** HRTEM image of (a) Al_2_O_3_ nanoneedle, (b) Al_2_O_3_ micro-needle, (c) Al_2_O_3_ spherical catalysts.

**Supplementary Figure 7 |** EDX mapping of Al_2_O_3_ nanoneedle, Al_2_O_3_ micro-needle and Al_2_O_3_ spherical catalysts.

**Supplementary Figure 8 |** N_2_ adsorption–desorption isotherms of the Al_2_O_3_ nanoneedle, Al_2_O_3_ micro-needle and Al_2_O_3_ spherical catalysts.

**Supplementary Figure 9 |** The pore size distribution curves for the samples from Al_2_O_3_ nanoneedle, Al_2_O_3_ micro-needle and Al_2_O_3_ spherical catalysts.

**Supplementary Figure 10 |** NH_3_-TPD profiles of the Al_2_O_3_ nanoneedle, Al_2_O_3_ micro-needle and Al_2_O_3_ spherical catalysts.

**Supplementary Figure 11 |** Py-IR spectra of the Al_2_O_3_ nanoneedle, Al_2_O_3_ micro-needle and Al_2_O_3_ spherical catalysts at 100 °C desorption temperature.

**Supplementary Figure 12 |** XPS spectra of O 1s of the Al_2_O_3_ nanoneedle, Al_2_O_3_ micro-needle and Al_2_O_3_ spherical catalysts.

**Supplementary Figure 13 |** XPS spectra of Al 2p of the Al_2_O_3_ nanoneedle, Al_2_O_3_ micro-needle and Al_2_O_3_ spherical catalysts.

**Supplementary Figure 14 |** CF_4_-TPD of the Al_2_O_3_ nanoneedle, Al_2_O_3_ micro-needle and Al_2_O_3_ spherical catalysts.

**Supplementary Figure 15 |** The details for *in situ* temperature monitoring under true fixed-bed reaction conditions.

**Supplementary Figure 16 |** Thermal infrared thermography of (a) Al_2_O_3_ nanoneedle (b) Al_2_O_3_ micro-needle and (c) Al_2_O_3_ spherical catalysts at 540 °C.

**Supplementary Figure 17 |** Thermal infrared thermography of Al_2_O_3_ nanoneedle, Al_2_O_3_ micro-needle and Al_2_O_3_ spherical catalysts from 75 to 580 °C.

**Supplementary Figure 18 |** Quantitative correlation between catalyst curvature and localized thermal enhancement.

**Supplementary Figure 19 |** (a) In situ DRIFTS associated with OH consumption during CF_4_ catalytic hydrolysis by Al_2_O_3_ nanoneedle catalyst (b) Al_2_O_3_ micro-needle and (c) Al_2_O_3_ spherical catalyst within the temperature range of 100 °C to 580 °C.

**Supplementary Figure 20 |** (a) In situ DRIFTS of CF_4_ catalytic hydrolysis by Al_2_O_3_ nanoneedle catalyst (b) Al_2_O_3_ micro-needle and (c) Al_2_O_3_ spherical catalyst within the temperature range of 580 °C.

**Supplementary Figure 21 |** (a) In situ DRIFTS of CF_4_ catalytic hydrolysis by Al_2_O_3_ nanoneedle catalyst (b) Al_2_O_3_ micro-needle catalyst and (c) Al_2_O_3_ spherical catalyst within the temperature range of 580 °C.

**Supplementary Figure 22 |** The stability test of the Al_2_O_3_ nanoneedle, Al_2_O_3_ micro-needle and Al_2_O_3_ spherical catalysts at 560 °C. (Reaction condition: 2500 ppm of CF_4_ and 10 % of H_2_O, balanced with Ar, total flow rate of 33.3 mL min^-1^, and weight hourly space velocity (WHSV) of 1000 mL g^-1^ h^-1^).

**Supplementary Table 1 |** BET results of Al_2_O_3_ nanoneedle, Al_2_O_3_ micro-needle and Al_2_O_3_ spherical catalysts.

| **Samples** | **SA (m^2^ g^-1^)** | **PV (cm^3^ g^-1^)** | **APR (nm)** |
| --- | --- | --- | --- |
| Al_2_O_3_ Nanoneedle | 259.708 | 0.632 | 9.729 |
| Al_2_O_3_ Micro-needle | 248.177 | 0.653 | 10.520 |
| Al_2_O_3_ Spherical | 268.559 | 0.667 | 9.828 |

**Supplementary Table 2 |** Turnover frequency of the CF_4_ decomposition at 500 °C.

| **Sample** | **surface Al_Ⅲ_ *^a^* (μmol)** | **CF_4_ decomposition *^b^* (%)** | **TOF *^c^* (10^-3^ s ^-1^)** |
| --- | --- | --- | --- |
| Al_2_O_3_ Nanoneedle | 6.037 | 27.32 | 2.84 |
| Al_2_O_3_ Micro-needle | 7.246 | 19.43 | 1.66 |
| Al_2_O_3_ Spherical | 8.455 | 14.73 | 1.08 |

a Determined by the NH_3_-TPD and Py-IR result.

b CF_4_ decomposition at 500 °C (2.0 g catalyst, 2500 ppm of CF_4_ and 10% of H_2_O balanced with Ar, 33.3 mL min^-1^).

c Turnover frequency of the CF_4_ decomposition at 500 °C.

Calculation equation: TOF=$\frac{\text{[CF}_{\text{4}}\text{ flow rate (μmol }\text{s}^{\text{-1}}\text{)]×[}\text{CF}_{\text{4}}\text{ decomposition]}}{\text{surface }\text{Al}_{\text{III}}}$

**Supplementary Table 3 |** Model parameters of COMSOL simulations.

| **Designation** | **Value** |
| --- | --- |
| $k_{iso,\gamma-{Al}_{2}O_{3}}$ | $11.8 W m^{-1}K^{-1}$ |
| $n_{sea urchin}$ | $5.128{*10}^{13} {kg}^{-1}$ |
| $n_{spherical}$ | $5.396{*10}^{13} {kg}^{-1}$ |
| $M_{{Al}_{2}O_{3}}$ | $0.10196 kg {mol}^{-1}$ |
| $M_{H_{2}O}$ | $0.0180153 kg {mol}^{-1}$ |
| $r_{spherical}$ | $1256 nm$ |
| $r_{tip}$ | $563 nm$ |
| $\rho_{bulk sea urchin}$ | $180.6 kg m^{-3}$ |
| $\rho_{bulk spherical}$ | $205.4 kg m^{-3}$ |
| $C_{p,air}$ | $1.004 J g^{-1} K^{-1}$ |
| $r_{reactor}$ | $1 cm$ |
| $\varepsilon_{sea urchin}$ | $0.763$ |
| $\varepsilon_{spherical}$ | $0.7924$ |
| $T_{0}$ | $853 K$ |
| *P_0_* | *1 atm* |
